# *Notes from the Field*: Absence of Asymptomatic Mumps Virus Shedding Among Vaccinated College Students During a Mumps Outbreak — Washington, February–June 2017

**DOI:** 10.15585/mmwr.mm6647a5

**Published:** 2017-12-01

**Authors:** Jesse Bonwitt, Vance Kawakami, Adam Wharton, Rachel M. Burke, Neil Murthy, Adria Lee, BreeAnna Dell, Meagan Kay, Jeff Duchin, Carole Hickman, Rebecca J. McNall, Paul A. Rota, Manisha Patel, Scott Lindquist, Chas DeBolt, Janell Routh

**Affiliations:** ^1^Epidemic Intelligence Service, Division of Scientific Education and Professional Development, CDC; ^2 ^Office of Communicable Disease Epidemiology, Washington State Department of Health; ^3^Public Health—Seattle & King County, Washington; ^4^National Center for Immunization and Respiratory Diseases, CDC; ^5^Department of Biomedical and Diagnostic Sciences, University of Tennessee, Knoxville; ^6^University of Washington, Seattle.

On February 8, 2017, a suspected case of mumps in a member of a fraternity or sorority at the University of Washington, Seattle campus (UW) was reported to Public Health—Seattle & King County (PHSKC). Additional confirmed and probable mumps cases were subsequently identified among UW students and staff members according to the national case definition.* By July 19, 2017, a total of 42 (16 confirmed and 26 probable) mumps cases were reported among UW students and associated community members, with symptom onset February 6–June 4 (Figure).

**FIGURE:**
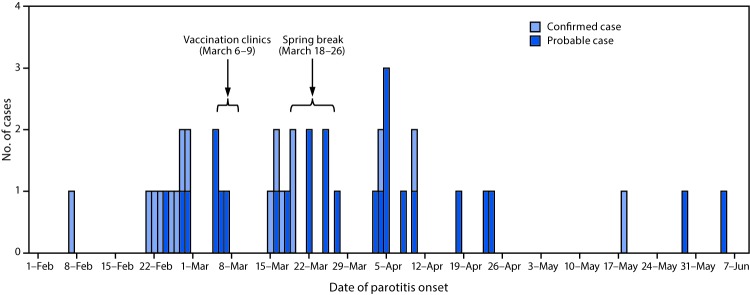
Number of confirmed and probable cases of mumps among fraternity and sorority members and associated community members, by date of parotitis onset — University of Washington, February–June 2017

Among the 42 cases, 32 (76%) occurred in UW fraternity and sorority members. Of these, 12 (37.5%) were confirmed cases, and 20 (62.5%) were probable cases. Cases occurred in residents in 20 (38.5%) of 52 fraternity and sorority houses that lodged 2,259 (48.6%) of 4,646 total fraternity and sorority members on the UW campus (42,000 students). All mumps patients had received ≥2 documented doses of measles-mumps-rubella (MMR) vaccine, as is currently recommended (*1*); 2-dose MMR coverage among all UW students exceeded 99%. Genotyping of viral isolates from four patients with confirmed mumps identified genotype G in all four, and molecular sequencing demonstrated differences between circulating strains at UW and a concurrent community outbreak in Washington.

On the basis of CDC guidance, PHSKC recommended an additional dose of MMR vaccine to protect fraternity and sorority members who were subject to potential mumps exposure (*2*). During March 6–9, the week before spring break, PHSKC administered 235 doses of MMR vaccine to members of the eight fraternity and sorority houses reporting the highest number of cases (Figure); the vaccination clinics were open to members of other fraternity and sorority houses.

Previous studies have suggested that mumps might be propagated by vaccinated persons with nonspecific symptoms or asymptomatic infection (*3*). Before licensure of mumps vaccine in 1967, 15%–27% of mumps infections were asymptomatic (*2*). How vaccination modifies clinical signs and symptoms of mumps is unknown (*2*). The prevalence of asymptomatic infection has not been assessed in the postvaccination era.

To assess the presence, prevalence, symptoms, and associated risk factors of asymptomatic mumps virus shedding in vaccinated persons, PHSKC, Washington State Department of Health, and CDC recruited a convenience sample of students at each MMR vaccination clinic. Participants provided written consent, completed a symptom and risk factor questionnaire, and provided a bilateral buccal swab immediately before or after vaccination. The Washington State Institutional Review Board determined this project to be nonresearch and exempt from review. Buccal swabs were collected from 160 of the 161 student participants, who represented at least eight fraternities and sororities; 80 (49.7%) were male. Participants reported the following symptoms during the preceding month, none of which required hospitalization: fever (10, 6%), cough (55, 34%), sore throat (37, 23%), and swelling or pain of the parotid gland or jaw not attributable to dental problems (eight, 5%). Specimens were processed at CDC. All 160 buccal swabs were mumps-virus negative by real-time reverse transcription–polymerase chain reaction; positive control testing in the laboratory indicated ˃99% successful specimen collection and processing.

The majority of mumps cases in this outbreak occurred among fraternity and sorority members; other studies have demonstrated that close contact is required for mumps transmission to occur in a population with high mumps vaccination coverage (*4*,*5*). This evaluation found no laboratory evidence of asymptomatic mumps virus shedding. Limitations include timing of sample collection, which might have missed the period when viral shedding was highest among infected persons, and the lack of serologic testing to identify infected participants. Serial sampling of exposed persons might yield different results. Mumps outbreaks have increased in recent years in the United States; from 2015 through 2016, the proportion of outbreak-related cases increased from 63% to 78% (*6*). Further evaluations to better understand the prevalence of mumps virus shedding among vaccinated populations are needed to guide outbreak surveillance and control.
